# The Management of Bacillus Calmette-Guérin (BCG) Failure in High-Risk Non-muscle Invasive Bladder Cancer: A Review Article

**DOI:** 10.7759/cureus.40962

**Published:** 2023-06-26

**Authors:** Ahmed Kodera, Mahmoud Mohammed, Paul Lim, Omer Abdalla, Mohammed Elhadi

**Affiliations:** 1 Urology, Worcestershire Acute Hopsitals NHS Trust, Worcester, GBR; 2 Urology, Worcestershire Acute Hospitals NHS Trust, Worcester, GBR; 3 Surgical Oncology, South Egypt Cancer Institute, Assiut, EGY; 4 Urology, Worcestershire Acute Hospitals NHS Trust, Worcestershire, GBR; 5 Urology, Wirral University Hospital, Liverpool, GBR; 6 Urology, Dudley Group NHS Hospital, Bromsrgrove, GBR

**Keywords:** non-muscle invasive bladder cancer, bcg refractory, bladder sparing, management, non- muscle invasive bladder cancer, bcg failure

## Abstract

Non-muscle invasive bladder cancer (NMIBC) is a common urological malignancy, and bacillus Calmette-Guérin (BCG) therapy is the gold standard treatment in intermediate and high-risk groups. However, BCG failure occurs in a significant proportion of patients, emphasizing the need for effective alternative treatment modalities to address this burden. These treatments include immunotherapy, enhanced drug delivery, targeted therapy, device-assisted chemotherapy, vaccine therapy, and gene therapy, which show varying degrees of safety and efficacy. The objective of this review is to summarize the current evidence and ongoing research on these emerging therapies, offering insight into their potential for improving patient outcomes and quality of life. Although radical cystectomy remains the standard of care for high-risk NMIBC patients unresponsive to BCG, novel treatment modalities hold promise for the future management of this challenging patient population.

## Introduction and background

Bladder cancer is the sixth most common cancer worldwide, with non-muscle invasive bladder cancer (NMIBC) accounting for approximately 75% of all cases [1]. The standard treatment for high-risk NMIBC patients involves intravesical bacillus Calmette-Guérin (BCG) therapy, which is a type of immunotherapy that has been proven to be effective in reducing tumor recurrence and progression [2]. A meta-analysis of 24 published randomized controlled trials (RCTs) demonstrated a 27% reduction in the odds of progression with BCG treatment (6.4% of 2,880 patients with papillary tumors and 13.9% of 403 patients with carcinoma in situ (CIS), based on a median follow-up of 2.5 years [3].

The European Association of Urology (EAU) guideline endorses intravesical BCG as the standard treatment for high-risk NMIBC following transurethral resection of a bladder tumour [3]. Similarly, the National Institute for Health and Care Excellence (NICE) guideline advocates intravesical BCG for high-risk NMIBC [4]. However, approximately 30% to 40% of patients receiving BCG treatment will experience failure, resulting in an elevated risk of recurrence and progression [5,6]

Radical cystectomy (RC) remains the gold-standard treatment option for BCG non-responders, offering a reasonable quality of life outcome and improved disease-specific survival if performed within two years of the initial BCG therapy [7,8]. Nonetheless, this may not be feasible for patients with significant comorbidities, those who prefer bladder preservation, or those who are reluctant to undergo major surgery. Therefore, urologists must provide comprehensive counseling to patients about alternative treatment options. This review highlights the bladder preservation strategies available to those patients who are unfit or unwilling to undergo RC.

## Review

Methodology

This narrative review of the literature focused on bladder-sparing options for BCG-unresponsive patients. We conducted a search of MEDLINE via PubMed, ClinicalTrials.gov registry, and Web of Science databases to identify clinical trials and studies evaluating the management of BCG-refractory, BCG-unresponsive, and BCG-failure in NMIBC. The search, performed in March 2023, utilized search terms such as 'BCG', 'BCG refractory', 'BCG unresponsive', 'BCG failure', 'bladder cancer', and 'bladder sparing'. We also examined major urology journals and international and national urology guidelines. This review included all relevant guidelines and clinical studies published in English, excluding those focusing on RC.

Definition of BCG-unresponsive high-risk NMIBC

Bacillus Calmette-Guérin-unresponsive high-risk NMIBC is defined as patients who are unlikely to achieve benefit from additional BCG therapy and should, if eligible, be offered RC as the standard of care. Further BCG instillations in these patients are associated with an increased risk of progression [9].

The BCG-unresponsive tumors include all BCG-refractory tumors in addition to those who develop T1/Ta HG recurrence within six months of completion of adequate BCG exposure or develop CIS within 12 months of completion of adequate BCG exposure. Adequate BCG is defined as at least five of six doses of induction BCG and two doses of maintenance.

For clinical practice and trial inclusion criteria, it is important to define the high-risk group of patients with a risk of recurrence, which lies between BCG-naïve and BCG-unresponsive NMIBC. There are three subpopulations in this group, which are summarized in Table 1 [9]. 

**Table 1:** High-risk NMIBC between BCG-naïve and BCG-unresponsive NMIBC (International Bladder Cancer Group consensus) NMIBC: Non-muscle invasive bladder cancer, BCG: Bacillus Calmette-Guérin, CIS: Carcinoma in situ

BCG-resistant NMIBC	Failure to achieve a disease-free state at the third-month evaluation after initial BCG. Standard treatment would be transurethral resection of the tumor and continued with maintenance/re-induction BCG
Delayed relapse after inadequate BCG	This situation mainly applies to patients who did not have maintenance following induction BCG, particularly in centers affected by BCG shortages.
Delayed relapse after adequate BCG	Recurrence of high-grade disease outside of the BCG-unresponsive window (>6 months for Ta/T1, and >12 months for CIS). Since a high-grade relapse beyond 24 months after the last dose of BCG would probably respond well to BCG again, this disease state would include only patients with relapse up to 24 months.

Can we predict BCG failure?

Several factors have been identified as predictors of BCG failure in patients with NMIBC, which can be divided into two main categories: patient factors and tumor factors. Patient factors include gender, age, and occupation. Studies have shown that male patients tend to exhibit better immunological responses to intravesical treatment and BCG compared to female patients, who may fail BCG treatment even after multiple attempts [10,11]. However, the female gender has not been shown to negatively impact disease progression [12,13], and both genders have similar outcomes concerning disease progression. Age also plays a role in BCG therapy outcomes; elderly patients diagnosed with NMIBC tend to have poorer responses to BCG treatment and other intravesical therapies, possibly due to reduced immune response and poor response to interleukins [14]. Joudi et al. found that patients older than 80 years have a 39% cancer-free survival rate at a median follow-up of 24 months, compared to 61% in younger patients. Occupation may also influence BCG therapy outcomes, as smoking tobacco, a major risk factor for bladder cancer, and exposure to certain industrial substances, such as polycyclic aromatic hydrocarbons, are known to impact disease progression [15].

Tumor factors, such as the number of tumors, presence of CIS, tumor size, and histological variants, can also predict BCG therapy outcomes. Patients with high-risk NMIBC, such as those with high-grade tumors, T1 stage disease, or CIS, are more likely to experience BCG failure compared to patients with an intermediate risk [16]. Additionally, larger tumor size, multifocality, and lymphovascular invasion have been associated with an increased risk of BCG failure [17,18]. Patients with multiple tumors are at a higher risk of disease recurrence after BCG treatment, although this does not necessarily correlate with disease progression [19,20]. Carcinoma in situ is considered a poor prognostic and predictive factor for disease recurrence, and its presence is associated with worse outcomes. Some studies, however, have reported a worse prognosis in concurrent CIS and T1 tumors compared to primary CIS [16].

Histological variants also have a prognostic role in predicting BCG failure. Studies reporting BCG response to high-grade NIMBC are predominately carried out on patients with pure urothelial cancer. In a variant, histology NMIBC, intravesical treatment has shown inferior outcomes compared to conventional transitional cell cancer (TCC) of the bladder. As such, patients with mixed histologic features are poor candidates for bladder-sparing protocols and are best served with radical treatment options. With the exception of glandular differentiation, which shows a promising response to intravesical BCG [21].

Further research has suggested that genetic and molecular markers could also serve as predictors of BCG failure. For example, variations in the expression of immune response genes, such as those involved in the production of cytokines or interferons, may influence the efficacy of BCG therapy [22-24]. Additionally, the presence of certain molecular subtypes of bladder cancer, such as luminal and basal subtypes, has been associated with different responses to BCG treatment [25]. However, more studies are needed to validate these findings and establish their clinical utility in predicting BCG failure in NMIBC patients.

What are the treatment options for BCG failure?

The definition of a BCG-unresponsive disease state has only evolved in the past several years. Prior to that, there was heterogeneity in patients enrolled in clinical trials and inconsistency regarding the definition of BCG failure. Hence, there was a move towards standardization of trial design, including patient cohorts, trial endpoints, and the control arm. Representatives of the FDA suggested that patients with CIS and Ta/T1 disease should be separated into two patient cohorts in the NMIBC clinical trial design. This, unfortunately, led to a decrease in sample size, which in turn had a negative impact on trial feasibility [9].

One of the reasons behind the separation is the trial endpoint. The primary endpoint for CIS (±Ta/T1) patients was suggested to be the cure rate (CR), with CR duration as the key secondary endpoint. On the other end, the primary endpoint for patients with Ta/T1 disease without CIS would be the duration of event-free survival (EFS). Relevant events would include persistence of CIS (at three or six months), high-grade recurrence (Ta/T1/CIS), progression to muscle-invasive or metastatic disease, the institution of a new radical or systemic therapy, and death from any cause [9].

Patients in the control arm of a randomized trial should be offered the best-established therapy for their disease state. Since BCG is considered the standard of care in a treatment-naïve setting, currently and for the foreseeable future, it would be considered the standard of care to treat BCG-exposed high-risk NMIBC with additional BCG [26,27].

BCG re-challenge

Repeat BCG instillation showed improved response rates in 40% to 60% of patients who did not respond to the first course of BCG [28], and this approach has been recommended by the European Association of Urology (EAU) and International Consultation on Urological Diseases (ICUD) for patients with BCG-refractory disease [29]. However, further courses of BCG (after two or more BCG failures) are not recommended as tumor progression is very likely and the chance of success is estimated to be below 20% [30].

BCG plus interferon-α2β

Interferon-α (IFN-α) is an immune modulator with antiproliferative activity. The potential for synergistic antitumor activity of IFN-α and BCG justifies the rationale of combination therapy for patients who do not tolerate or respond to standard-dose BCG monotherapy. Some non-randomized studies have shown promise [31,32].

A national multicenter randomized phase II trial of the combination conducted by Joudi et al. included 1,007 patients and showed 45% disease-free survival at two years [31]. However, a subsequent systematic review of the role of the combination versus BCG alone found no clear evidence of the superiority of the combination over BCG in terms of recurrence or progression [33].

Intravesical chemotherapy

Gemcitabine

Gemcitabine is a deoxycytidine analog presenting broad-spectrum antitumor activity plus limited local toxicity when administered intravesically. Mohanty et al. assessed the efficacy of intravesical administration of gemcitabine in 35 patients with BCG failure. All patients received six consecutive weekly instillations of 2000 mg of gemcitabine in 50 ml of normal saline. After a mean follow-up of 18 months, 60% showed no recurrences, 31.4% presented with superficial recurrences, and 9% progressed into the muscle-invasive stage [34].

In addition, gemcitabine efficacy versus BCG repeat was tested in a multicenter prospective randomized phase II trial. Patients in the gemcitabine arm received intravesical instillations twice a week (2000 mg/50 ml) for six weeks followed by three consecutive weekly instillations at three, six, and 12 months. Patients in the BCG arm received a six-week induction course plus a maintenance course. Disease recurrence was 52.5% in the gemcitabine arm versus 87.5% in the BCG arm. Disease progression to muscle-invasive status was recorded in 33% of gemcitabine patients compared to 37.5% of BCG patients [35]. However, among patients failing BCG twice, a prospective trial named Southwest Oncology Group (SWOG) S0353 showed disappointing results with single-agent gemcitabine, as only 24% of patients remained disease-free at 12 months and only 21% at 24 months [36].

Docetaxel

Docetaxel is a taxane widely used in metastatic prostate cancer. In a study by Barlow et al., a total of 54 patients with BCG-refractory disease received six weekly instillations plus a single monthly maintenance dose for a total duration of one year. After a median follow-up of 39.1 months. One- and three-year recurrence-free survival (RFS) rates for the entire cohort were 40% and 25%, respectively. Of the 54 patients, 17 (24%) underwent RC at a median of 24 months of follow-up. Five-year disease-specific and overall survival rates were 85% and 71%, respectively [37].

Sequential Intravesical Gemcitabine and Mitomycin C

A retrospective analysis of patients who received sequential treatment with 1g of gemcitabine and 40 mg mitomycin C (MMC), was performed. A total of 47 patients with BCG failure were included. Gemcitabine was installed first, retained for 90 minutes, then drained, and the instillation of MMC followed.

In terms of results, complete response, one-year, and two-year RFS rates for all patients were 68%, 48%, and 38%, respectively; and after a median follow-up of 26 months, 10 patients underwent RC [38]. These results were subsequently validated in a 27-patient (24 BCG failure) retrospective study at the Mayo Clinic, where 37% remained disease-free at a median of 22 months of follow-up [39].

Sequential Intravesical Gemcitabine and Docetaxel

In a study by Steinberg et al., 45 patients with sequential intravesical treatment with gemcitabine (1 g) and docetaxel (37.5 mg in 50 ml) were evaluated. Treatment was successful in 66% of patients at first surveillance, 54% at 1 year, and 34% at two years after initiating induction. Ten patients underwent cystectomy [40].

In a multi-center retrospective study of combined gemcitabine-docetaxel on 276 BCG failure patients, the high-grade RFS at 24 months was over 50%, even among those with BCG-unresponsive disease. Monthly maintenance therapy for up to two years was noted to be associated with a significant outcome improvement at two years. Furthermore, only 16% required cystectomy, and only 4% had progression. However, no prospective trials have yet been reported with this therapy [41].

Triple Intravesical Therapy With Intravesical Cabazitaxel, Gemcitabine, and Cisplatin

In a phase I trial by DeCastro et al., 18 participants with BCG-unresponsive non-muscle invasive urothelial bladder cancer were recruited. All patients underwent a six-week induction regimen of sequentially administered cabazitaxel, gemcitabine, and cisplatin (CGC). A complete response was defined as no cancer on post-induction transurethral bladder tumor resection and negative urine cytology. There were no dose-limiting toxicities. Initial partial and complete response rates were 94% and 89%, respectively [42].

Electromotive drug administration

Electromotive drug administration (EMDA) is a device-assisted method of MMC intravesical instillation. It promotes drug uptake through electroporation, electro-osmosis, and iontophoresis. The device uses a catheter inserted into the bladder, two electrodes placed in the suprapubic area, and a generator. A single-center, single-arm phase II trial was carried out to investigate the efficacy and safety of EMDA-MMC. A total of 26 patients presenting with BCG-refractory, high-grade NMIBC were included. Patients were followed up for three years, and 61.5% of the patients could avoid RC. Progression into a muscle-invasive disease was reported in 15.4% of patients. Treatment was tolerable in only three patients with severe systemic reactions [43]. The effectiveness of BCG vs. sequential BCG instillations and EMDA-MMC instillations in preventing the recurrence and progression of high-risk NMIBC is under investigation in a phase III trial (NCT03664869).

Chemohyperthermia

Chemohyperthermia is a way to improve MMC efficacy through intravesical hyperthermia of bladder urothelium. The most common system used is the Synergo HT system (Medical Enterprises, Amsterdam, Netherlands). The target bladder temperature (44 to 48 degrees) is achieved by an intravesical microwave applicator. This increase in bladder wall temperature facilitates the penetration of MMC into the urothelium and also has a cytotoxic effect on cancer cells.

The UK bladder cancer trial (HYMN) aims to further define the role of thermochemotherapy. It is a randomized phase III trial designed to determine the effectiveness of hyperthermia in combination with MMC versus BCG or standard therapy as a second-line treatment for patients with recurrent NMIBC following induction or maintenance therapy with BCG. Two-year disease-free survival was 35% in the radiofrequency-induced thermo-chemotherapy effect (RITE) arm versus 41% in the control arm [44].

An alternative system for chemohyperthermia is the hyperthermic intravesical chemotherapy (HIVEC) system, which rewarms and circulates hot mitomycin in the bladder. The difference is that in HIVEC, MMC is extravesical heated by the Combat BRS system (Combat Medical Ltd., St. Albans, Hertfordshire, UK) and recirculated, maintaining a high bladder temperature. In a retrospective, multicenter study with no comparative arm, the efficacy of HIVEC was tested. The two-year RFS rate was 19.9% and 43.7% in patients with and without CIS, respectively (p=0.52). Fifteen patients (12.9%) experienced progression to muscle-invasive bladder cancer with no significant difference between patients with and without CIS (two-year progression-free survival (PFS) rate = 1.8% vs. 88.8%, p=0.32) [45].

Vicinium

Oportuzumab monatox (vicinium) is a recombinant fusion protein composed of an epithelial cell adhesion molecule (EpCAM)-specific antibody fragment linked with a pseudomonas exotoxin. The antigen is overexpressed in tumor cells [46]. In BCG-unresponsive CIS patients, phase I and II trials (NCT02449239) demonstrated safety and effectiveness, with a complete response (CR) rate of 40% and 16% at three and 12 months, respectively, with only 4% grade 3 side effects [47].

Gene therapy

Nadofaragene Firadenovec (Adstiladrin)

Nadofaragene firadenovec is an intravesical human IFN-α2β gene-mediated therapy that delivers the IFN-α2β gene to increase IFN-α2β expression. In a phase III study, a CR rate of 53.4% at three months was achieved in patients with CIS, with 24.3% remaining free of high-grade recurrence at one year. In patients with high-grade Ta/T1 alone, 72.9% and 43.8% were free from recurrence at three and 12 months, respectively. Overall, responses were durable for one year [48].

CG0070

The CG0070 is an oncolytic serotype 5 adenovirus that targets the downregulated retinoblastoma tumor suppressor (Rb). It demonstrated antitumor activity in preclinical bladder cancer models [49]. The efficacy of CG0070 in patients with BCG failure was evaluated by Packiam et al. in a phase II/III study. In terms of efficacy, intravesical administration of CG0070 achieved complete response rates in 44%, 30%, and 23% of patients in six, 12, and 18 months, respectively [50]. The combination of CG0070 + pembrolizumab is under investigation in the Core-001 trial.

Vaccine therapy

Ongoing trials on vaccine therapy are summarized in Table 2 [51-53].

**Table 2:** Vaccine therapy IM: Intramuscular, IV: Intravenous, ID: Intradermal, BCG: Bacillus Calmette-Guérin, DFS: Disease-free survival

Vaccine	Clinical Trial Number	Administration Route	Study arm(s)	Mechanism of action	Primary Outcome
PANVAC	NCT02015104	IM	BCG plus PANVAC versus BCG monotherapy	PANVAC is a recombinant poxviral vaccine. The mechanism of action involves the activation of CD4 and CD8 antigen-specific response as it recognizes tumor more associated antigens, carcinoembryonic antigens, and mucin-1.	DFS
Alt-803	NCT01625260	IV	Single-arm intervention with Alt-803 plus gemcitabine	Alt-803 is a recombinant fusion protein that acts as an IL-15 superagonist complex and thus involves in the development and activation of natural killer cells and CD-8 cells	Recommended dose level, safety and tolerability of Alt-803
HS-410	NCT02010203	ID	Phase 1: BCG-relapsing induction therapy followed by one-year disease-free low-dose ID HS-410 monotherapy; Phase 2: ID placebo or low dose or high dose HS-410 in combination with induction and maintenance BCG	An allogeneic cell line, selected for high expression from a series of bladder tumor antigens, and transfected with gp96-Ig. Secretion of gp96 heat shock protein stimulates CD-8 cytotoxic cell production.	Safety, tolerability, and one-year DFS

Photodynamic therapy

Photodynamic therapy (PDT) uses the interaction between absorbed light and retained photosensitizing agents to destroy cancer cells through the production of reactive oxygen species. Early trials have shown the efficacy of PDT in BCG-refractory CIS and BCG-failed superficial TCC with a CR in 3 months in 58% and 45% of patients, respectively. However, severe local side effects were experienced due to the lack of specificity of photosensitizers [54,55]. A phase I study for TLD1433 has been completed, and a larger phase II trial in the BCG-unresponsive setting is underway (NCT03945162).

Immunotherapy

The emergence of checkpoint inhibition (CPI) therapies in the treatment of breast cancer has also opened up new opportunities in NMIBC. Initially, programmed cell death ligand-1 (PD-L1) inhibitors were only trialed in metastatic breast cancer. The encouraging outcome widened their spectrum of use to NMIBC.

Pembrolizumab

The KEYNOTE-057, an open-label, single-arm, multicentre phase II study, evaluated the use of a PD-L1 inhibitor (pembrolizumab) in BCG-unresponsive NMIBC. A total of 101 patients were enrolled and received pembrolizumab 200 mg intravenously every three weeks for up to 24 months. The primary endpoint was the CR rate. The median follow-up was 36·4 months. Around 41% of patients with BCG failure had a CR at 3 months. Grade 3 or 4 treatment-related adverse events occurred in 13% of the patients; serious treatment-related adverse events occurred in eight (8%) patients. And this shows that pembrolizumab monotherapy was well tolerated and had promising results [56]. In January 2020, the FDA approved the use of pembrolizumab for the indication of patients with BCG-refractory CIS with or without a papillary tumor that is not suitable for RC or those declining it [57].

Atezolizumab

The SWOG S1605 (NCT02844816), a single-arm phase II trial, evaluated the CPI of atezolizumab in patients with BCG-unresponsive high-risk NMIBC who were ineligible for or refused RC. Atezolizumab was given intravenously at a dosage of 1200 mg every three weeks for one year. The patients were divided into two cohorts according to the primary T-stage: CIS and TaT1/HG tumors. The primary endpoint was the pathological CR rate at six months, as defined by a mandatory biopsy. From the CIS cohort (n=74), 27% of patients had a six-month CR and a median duration of response (DoR) of CR of 15.4 months. The 18-month EFS was 47.1%, 17%, and 29% in the TaT1/HG cohort, the CIS cohort, and the overall population, respectively. Three patients progressed to muscle-invasive disease at three (n=2) and at 22 months (n = 1). Two patients developed metastatic disease without bladder recurrence at 17 and 31 months [58].

## Conclusions

As RC remains the gold standard for patients with NMIBC facing BCG failure, there is growing interest in establishing novel therapeutic methods that combine comparable oncological results with the benefit of preserving the bladder. Treatment choice is mainly influenced by accessibility to treatment, patient preference, and cost-related factors.

For patients in whom BCG has once failed, a repeat course of BCG or BCG plus interferon appears to be a reasonable clinical practice. However, after two or more BCG failures, especially in patients with earlier relapses or cancer persistence, combined intravesical chemotherapy yields better results than a single agent, with an impressive one- and two-year CR rate. In the same setting, intravesical nadofaragene firadenovec is effective, with a good safety profile and ease of delivery. Despite the potential benefit of tackling micrometastatic disease and the durable CR, immunotherapy is unlikely to be a first-line treatment, mainly due to cost and partly due to immune-related systemic side effects.

Owing to the lack of large-scale clinical trials, guidelines for managing NMIBC have not yet endorsed recommendations for salvage agents following established BCG failure. As such, the cost of salvage therapy may not be approved by insurance companies or public health systems such as the NHS, subsequently limiting options to only drugs listed in treatment guidelines. Thus, whilst more robust data is required to develop guidelines, available salvage treatment options should be discussed with patients, and recruitment for clinical trials needs to continue. Results from ongoing trials will provide us with more insight into many of the existing regimens, and potentially new drugs will soon be available for this group of patients.
